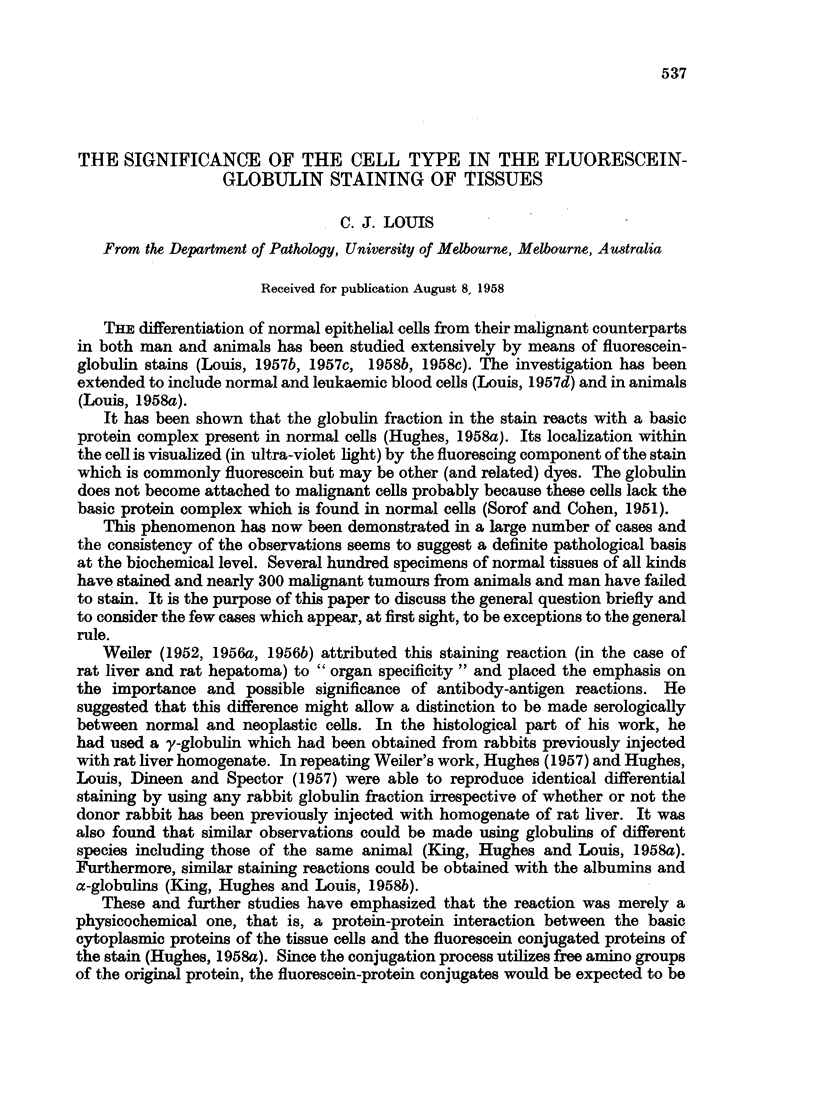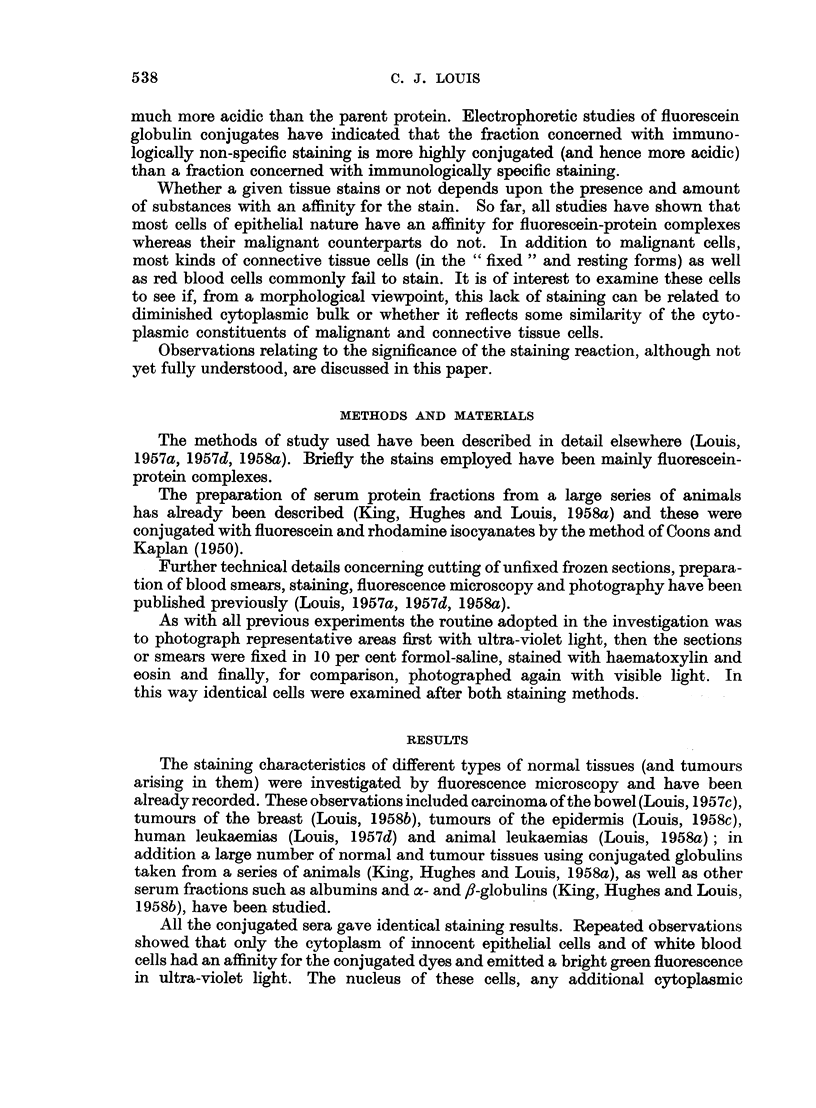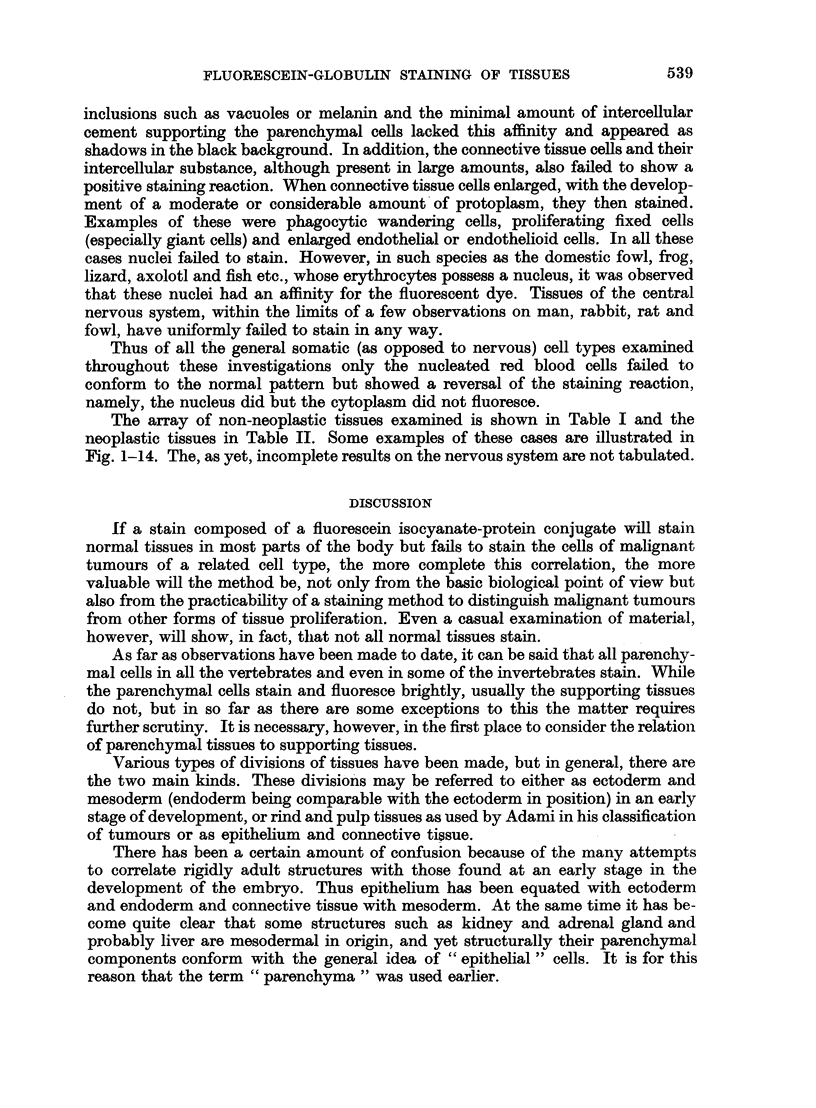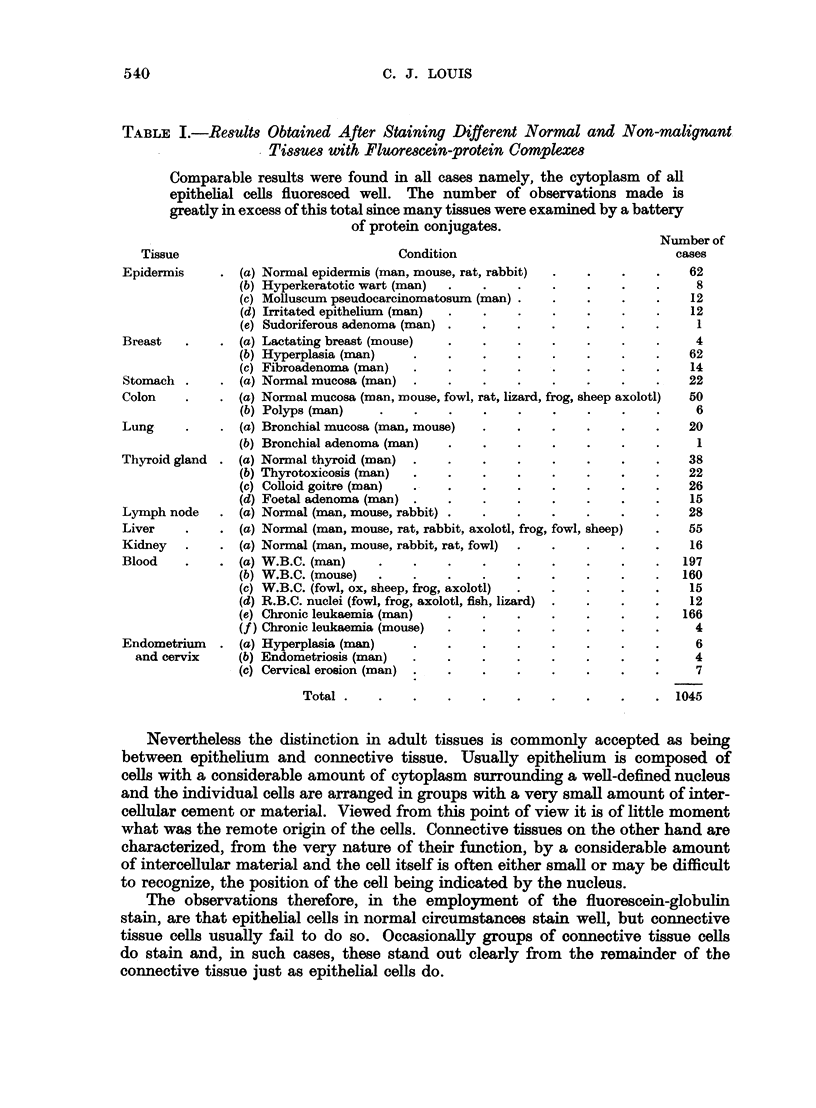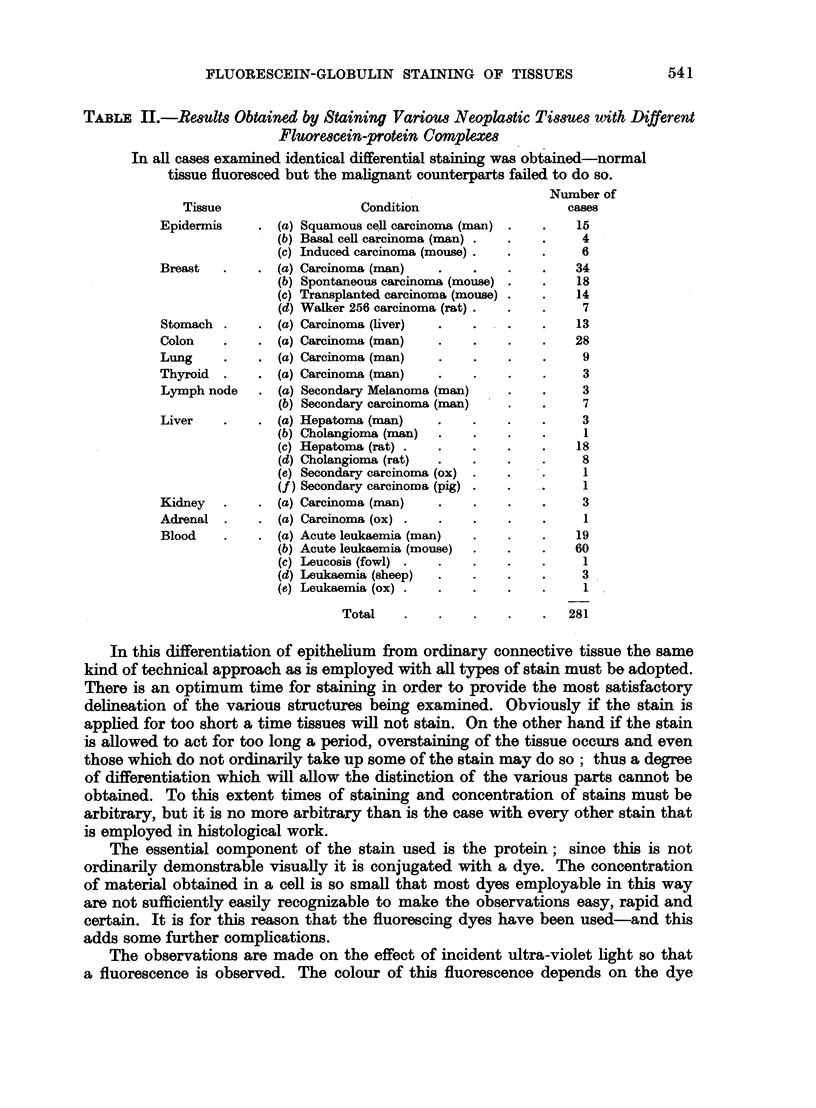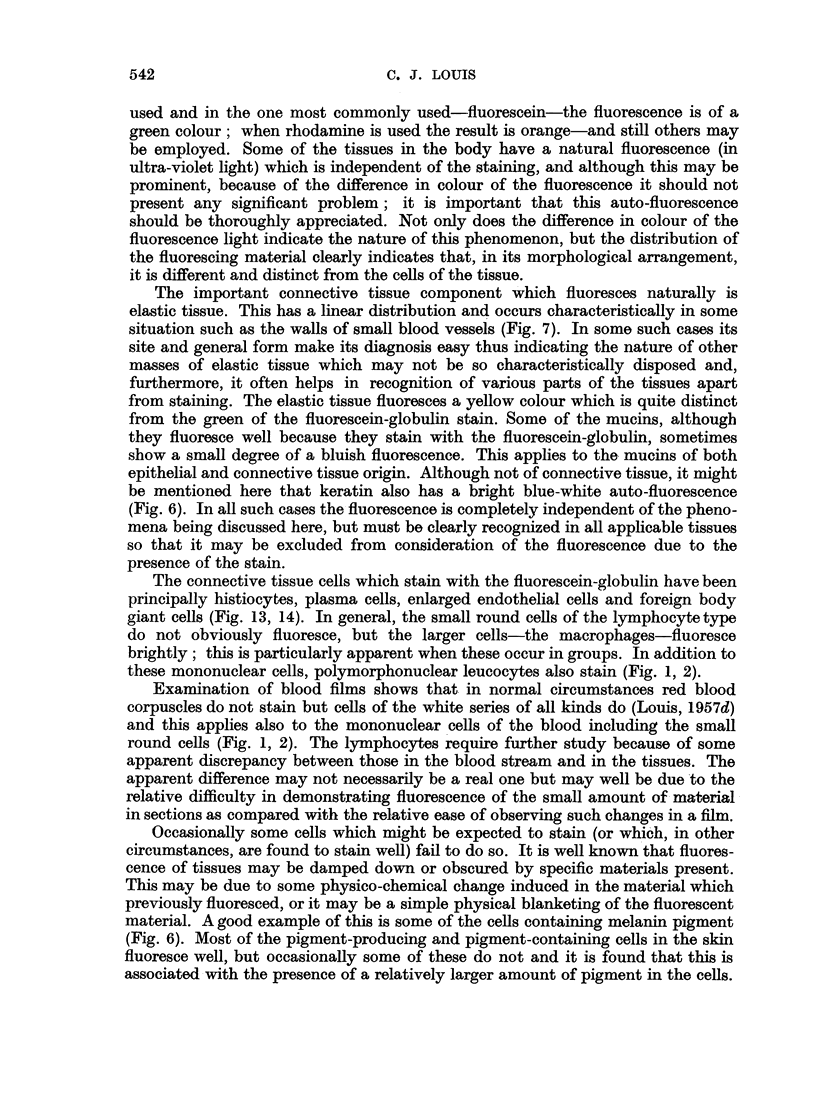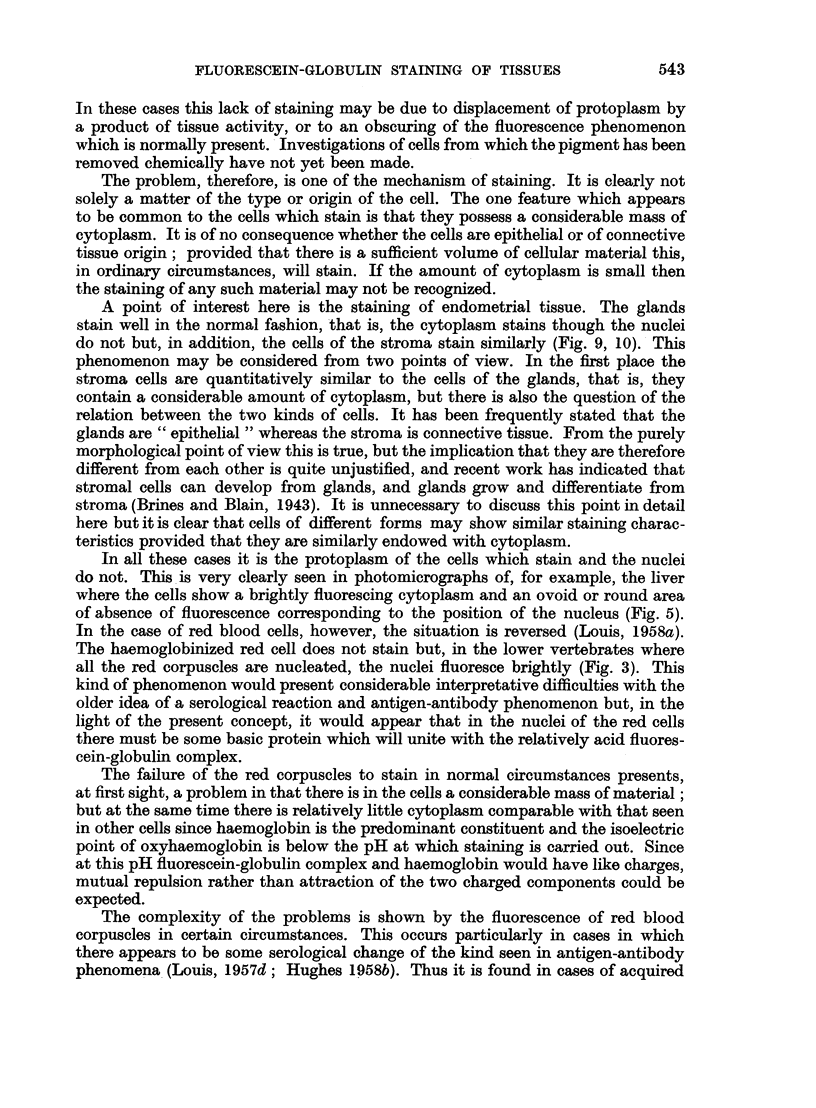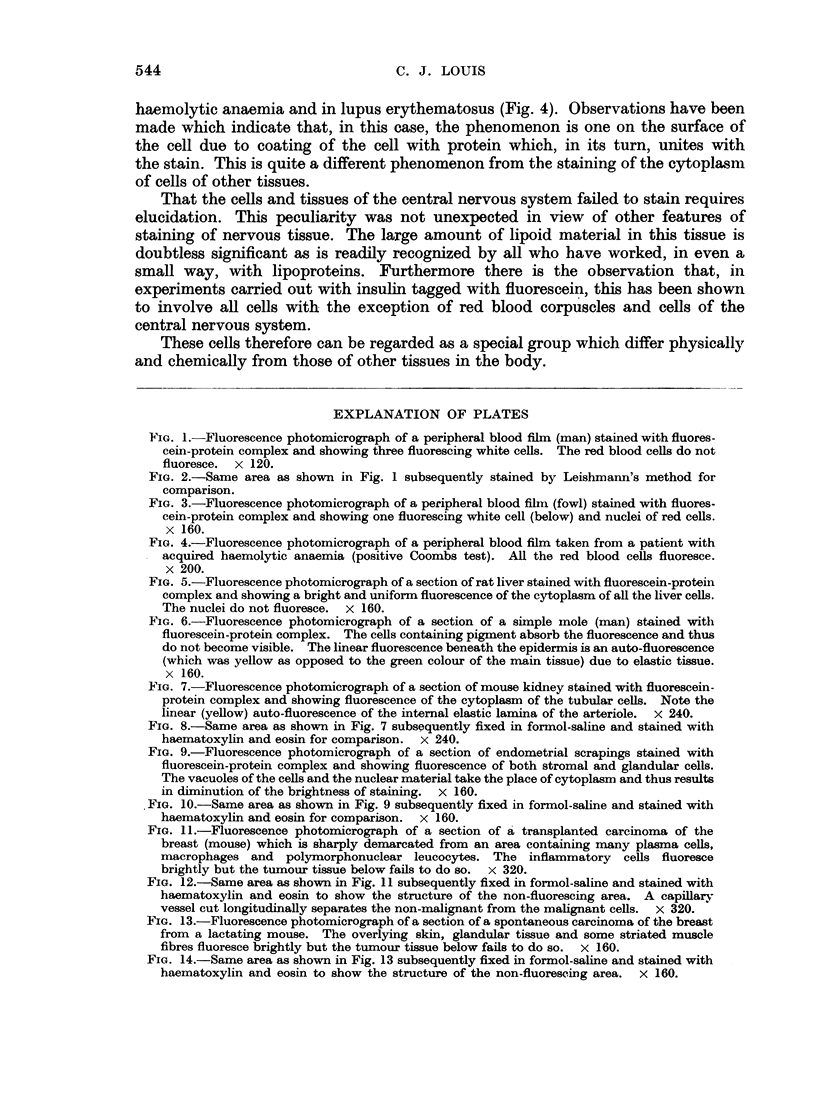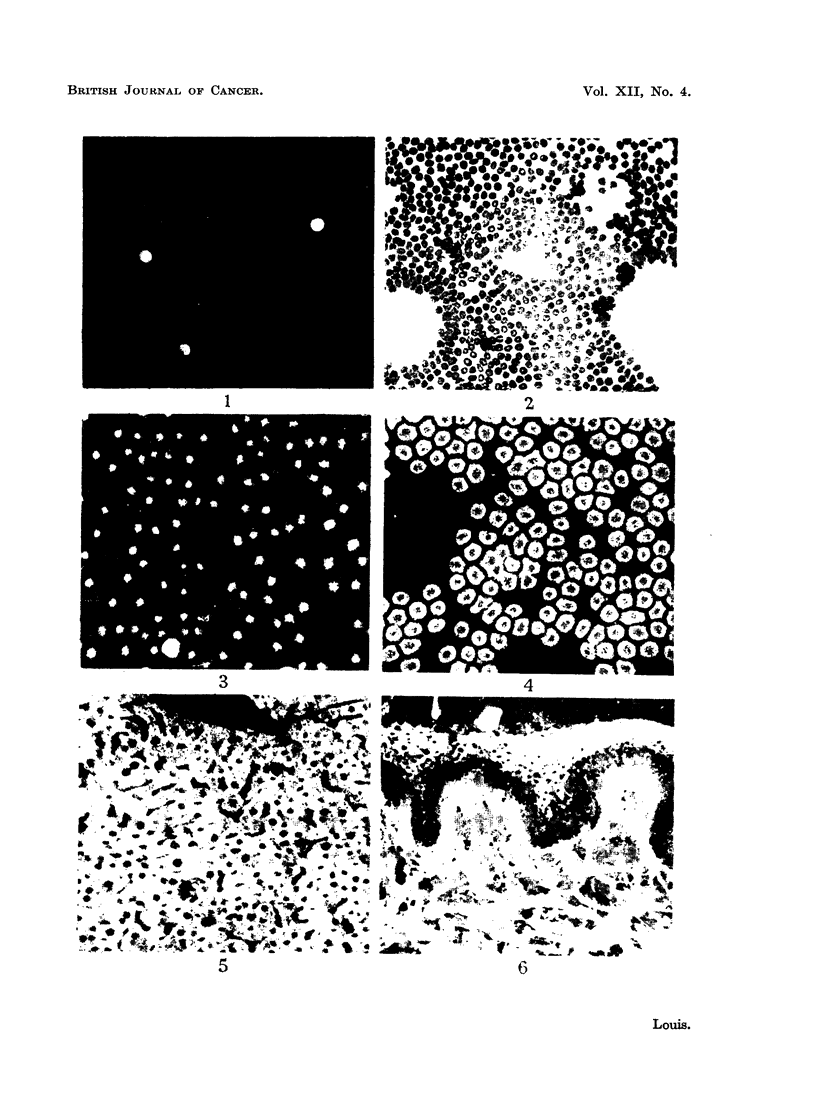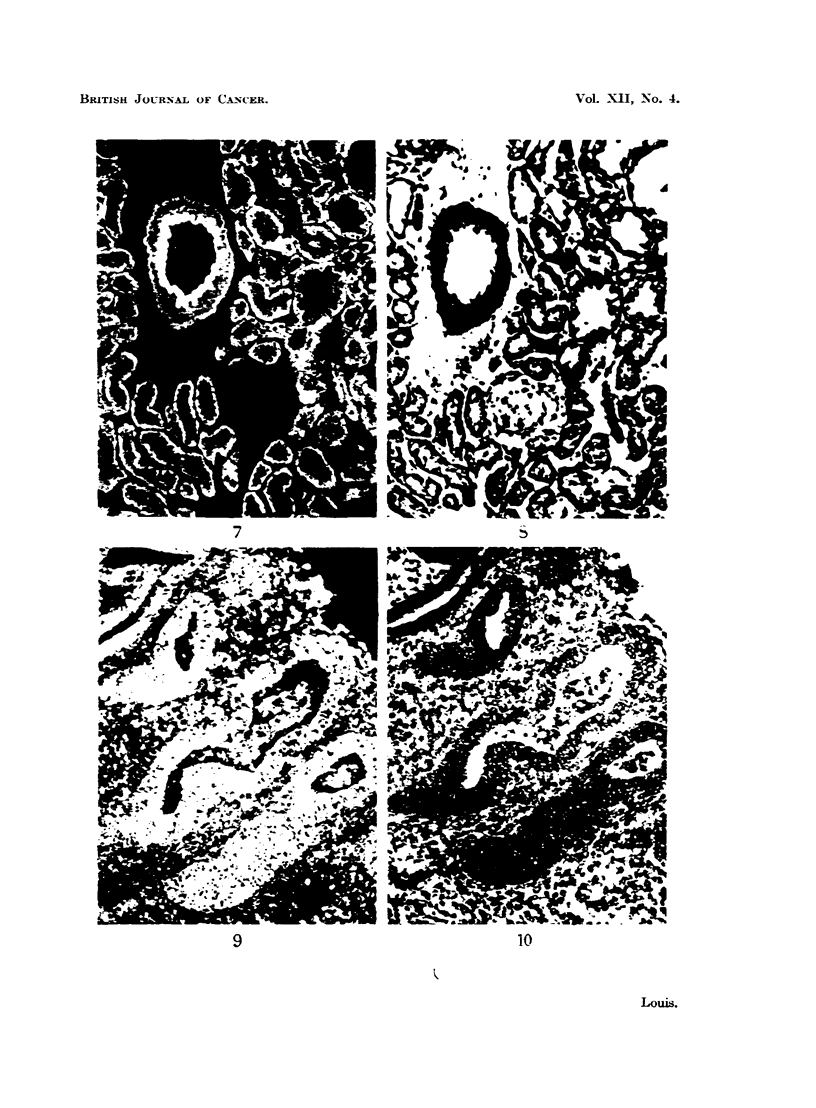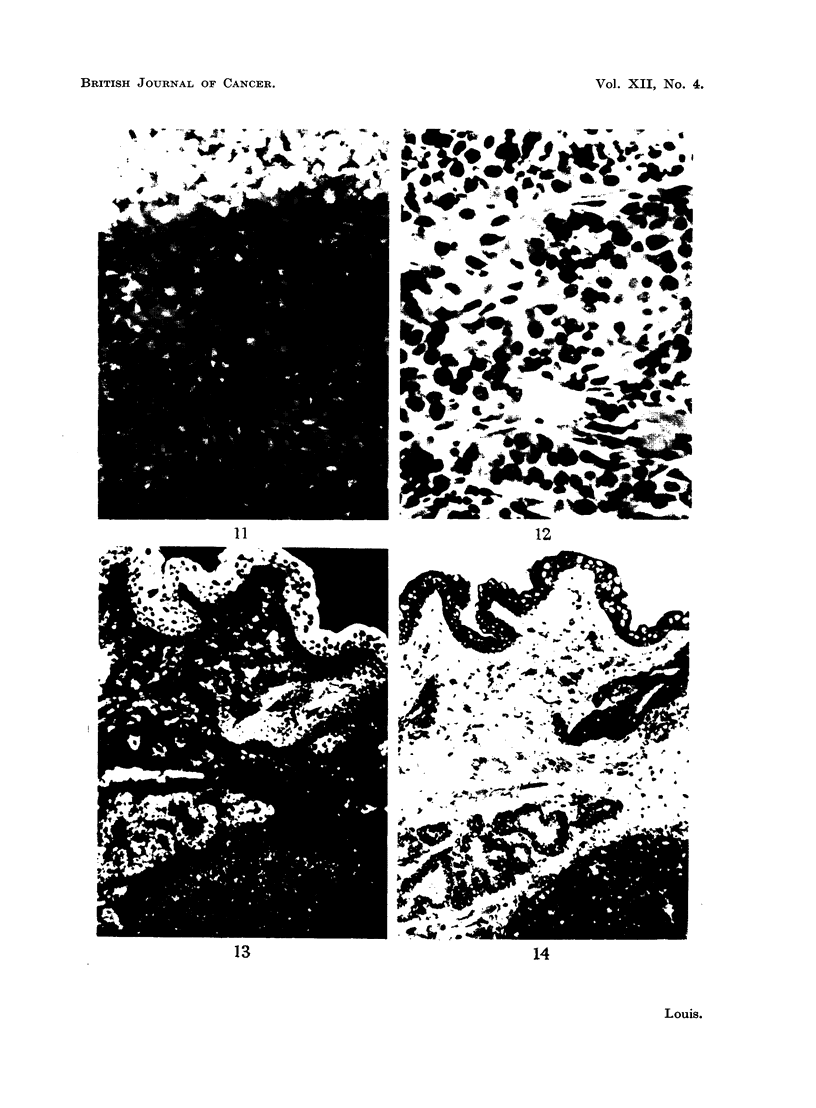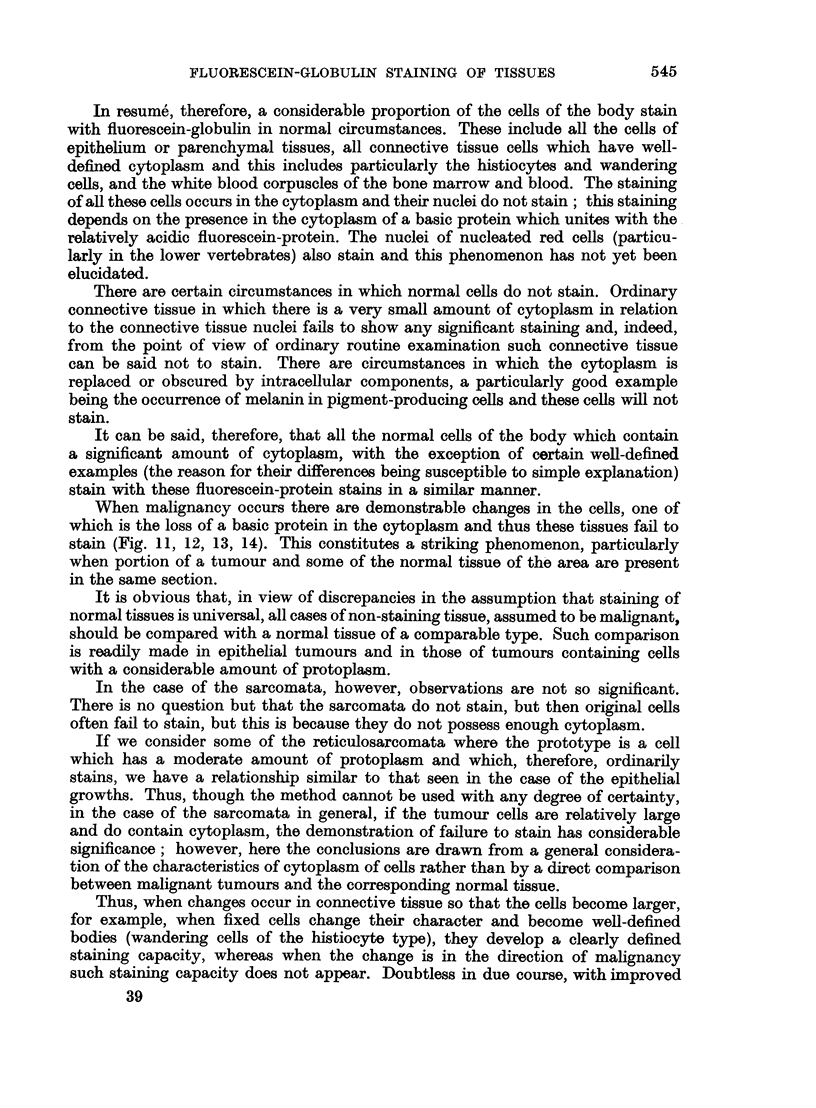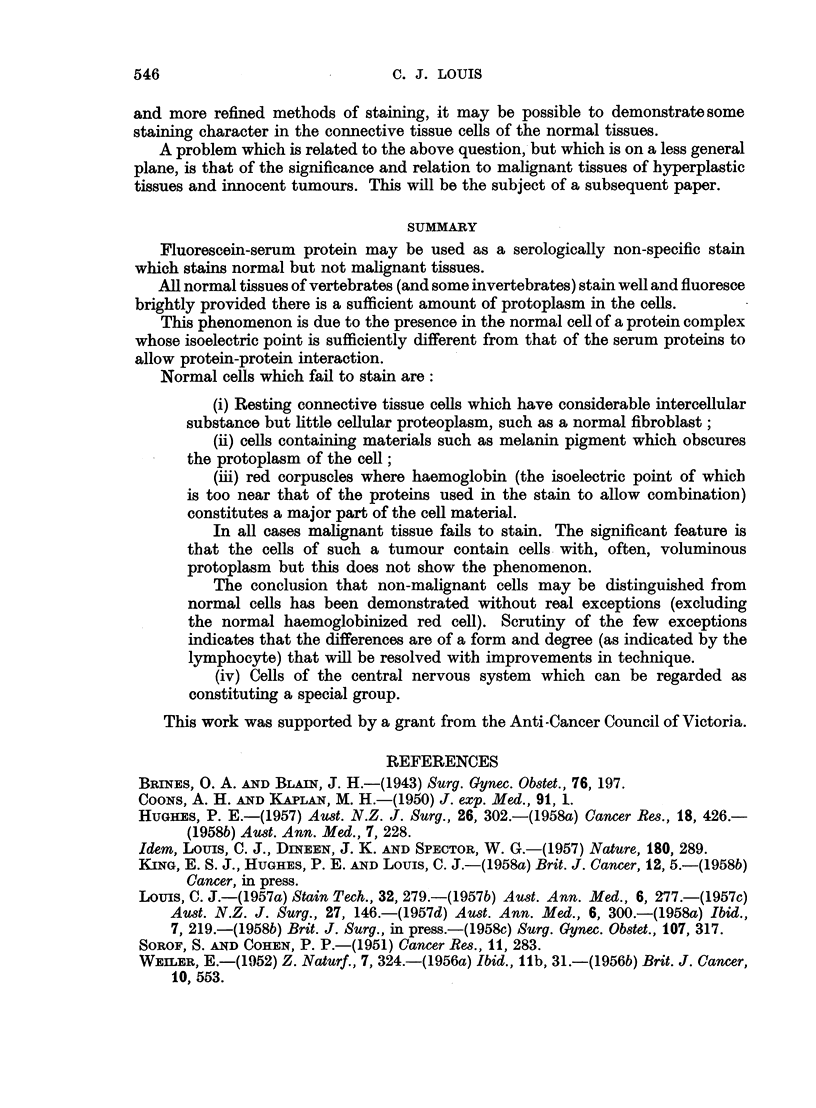# The Significance of the Cell Type in the Fluorescein-Globulin Staining of Tissues

**DOI:** 10.1038/bjc.1958.62

**Published:** 1958-12

**Authors:** C. J. Louis

## Abstract

**Images:**


### THE SIGNIFICANCE OF THE CELL TYPE IN THE FLUORESCEIN-GLOBULIN STAINING OF TISSUES

C. J. LOUIS

*From the Department of Pathology, University of Melbourne, Melbourne, Australia*

Received for publication August 8, 1958

THE differentiation of normal epithelial cells from their malignant counterparts in both man and animals has been studied extensively by means of fluorescein-globulin stains (Louis, 1957*b*, 1957*c*, 1958*b*, 1958*c*). The investigation has been extended to include normal and leukaemic blood cells (Louis, 1957*d*) and in animals (Louis, 1958*a*).

It has been shown that the globulin fraction in the stain reacts with a basic protein complex present in normal cells (Hughes, 1958*a*). Its localization within the cell is visualized (in ultra-violet light) by the fluorescing component of the stain which is commonly fluorescein but may be other (and related) dyes. The globulin does not become attached to malignant cells probably because these cells lack the basic protein complex which is found in normal cells (Sorof and Cohen, 1951).

This phenomenon has now been demonstrated in a large number of cases and the consistency of the observations seems to suggest a definite pathological basis at the biochemical level. Several hundred specimens of normal tissues of all kinds have stained and nearly 300 malignant tumours from animals and man have failed to stain. It is the purpose of this paper to discuss the general question briefly and to consider the few cases which appear, at first sight, to be exceptions to the general rule.

Weiler (1952, 1956*a*, 1956*b*) attributed this staining reaction (in the case of rat liver and rat hepatoma) to " organ specificity " and placed the emphasis on the importance and possible significance of antibody-antigen reactions. He suggested that this difference might allow a distinction to be made serologically between normal and neoplastic cells. In the histological part of his work, he had used a $\gamma$-globulin which had been obtained from rabbits previously injected with rat liver homogenate. In repeating Weiler's work, Hughes (1957) and Hughes, Louis, Dineen and Spector (1957) were able to reproduce identical differential staining by using any rabbit globulin fraction irrespective of whether or not the donor rabbit has been previously injected with homogenate of rat liver. It was also found that similar observations could be made using globulins of different species including those of the same animal (King, Hughes and Louis, 1958*a*). Furthermore, similar staining reactions could be obtained with the albumins and $\alpha$-globulins (King, Hughes and Louis, 1958*b*).

These and further studies have emphasized that the reaction was merely a physicochemical one, that is, a protein-protein interaction between the basic cytoplasmic proteins of the tissue cells and the fluorescein conjugated proteins of the stain (Hughes, 1958*a*). Since the conjugation process utilizes free amino groups of the original protein, the fluorescein-protein conjugates would be expected to be much more acidic than the parent protein. Electrophoretic studies of fluorescein globulin conjugates have indicated that the fraction concerned with immunologically non-specific staining is more highly conjugated (and hence more acidic) than a fraction concerned with immunologically specific staining.

Whether a given tissue stains or not depends upon the presence and amount of substances with an affinity for the stain. So far, all studies have shown that most cells of epithelial nature have an affinity for fluorescein-protein complexes whereas their malignant counterparts do not. In addition to malignant cells, most kinds of connective tissue cells (in the " fixed " and resting forms) as well as red blood cells commonly fail to stain. It is of interest to examine these cells to see if, from a morphological viewpoint, this lack of staining can be related to diminished cytoplasmic bulk or whether it reflects some similarity of the cytoplasmic constituents of malignant and connective tissue cells.

Observations relating to the significance of the staining reaction, although not yet fully understood, are discussed in this paper.

### METHODS AND MATERIALS

The methods of study used have been described in detail elsewhere (Louis, 1957*a*, 1957*d*, 1958*a*). Briefly the stains employed have been mainly fluorescein-protein complexes.

The preparation of serum protein fractions from a large series of animals has already been described (King, Hughes and Louis, 1958*a*) and these were conjugated with fluorescein and rhodamine isocyanates by the method of Coons and Kaplan (1950).

Further technical details concerning cutting of unfixed frozen sections, preparation of blood smears, staining, fluorescence microscopy and photography have been published previously (Louis, 1957*a*, 1957*d*, 1958*a*).

As with all previous experiments the routine adopted in the investigation was to photograph representative areas first with ultra-violet light, then the sections or smears were fixed in 10 per cent formol-saline, stained with haematoxylin and eosin and finally, for comparison, photographed again with visible light. In this way identical cells were examined after both staining methods.

### RESULTS

The staining characteristics of different types of normal tissues (and tumours arising in them) were investigated by fluorescence microscopy and have been already recorded. These observations included carcinoma of the bowel (Louis, 1957*c*), tumours of the breast (Louis, 1958*b*), tumours of the epidermis (Louis, 1958*c*), human leukaemias (Louis, 1957*d*) and animal leukaemias (Louis, 1958*a*); in addition a large number of normal and tumour tissues using conjugated globulins taken from a series of animals (King, Hughes and Louis, 1958*a*), as well as other serum fractions such as albumins and $\alpha$- and $\beta$-globulins (King, Hughes and Louis, 1958*b*), have been studied.

All the conjugated sera gave identical staining results. Repeated observations showed that only the cytoplasm of innocent epithelial cells and of white blood cells had an affinity for the conjugated dyes and emitted a bright green fluorescence in ultra-violet light. The nucleus of these cells, any additional cytoplasmic inclusions such as vacuoles or melanin and the minimal amount of intercellular cement supporting the parenchymal cells lacked this affinity and appeared as shadows in the black background. In addition, the connective tissue cells and their intercellular substance, although present in large amounts, also failed to show a positive staining reaction. When connective tissue cells enlarged, with the development of a moderate or considerable amount of protoplasm, they then stained. Examples of these were phagocytic wandering cells, proliferating fixed cells (especially giant cells) and enlarged endothelial or endothelioid cells. In all these cases nuclei failed to stain. However, in such species as the domestic fowl, frog, lizard, axolotl and fish etc., whose erythrocytes possess a nucleus, it was observed that these nuclei had an affinity for the fluorescent dye. Tissues of the central nervous system, within the limits of a few observations on man, rabbit, rat and fowl, have uniformly failed to stain in any way.

Thus of all the general somatic (as opposed to nervous) cell types examined throughout these investigations only the nucleated red blood cells failed to conform to the normal pattern but showed a reversal of the staining reaction, namely, the nucleus did but the cytoplasm did not fluoresce.

The array of non-neoplastic tissues examined is shown in Table I and the neoplastic tissues in Table II. Some examples of these cases are illustrated in Fig. 1–14. The, as yet, incomplete results on the nervous system are not tabulated.

### DISCUSSION

If a stain composed of a fluorescein isocyanate-protein conjugate will stain normal tissues in most parts of the body but fails to stain the cells of malignant tumours of a related cell type, the more complete this correlation, the more valuable will the method be, not only from the basic biological point of view but also from the practicability of a staining method to distinguish malignant tumours from other forms of tissue proliferation. Even a casual examination of material, however, will show, in fact, that not all normal tissues stain.

As far as observations have been made to date, it can be said that all parenchymal cells in all the vertebrates and even in some of the invertebrates stain. While the parenchymal cells stain and fluoresce brightly, usually the supporting tissues do not, but in so far as there are some exceptions to this the matter requires further scrutiny. It is necessary, however, in the first place to consider the relation of parenchymal tissues to supporting tissues.

Various types of divisions of tissues have been made, but in general, there are the two main kinds. These divisions may be referred to either as ectoderm and mesoderm (endoderm being comparable with the ectoderm in position) in an early stage of development, or rind and pulp tissues as used by Adami in his classification of tumours or as epithelium and connective tissue.

There has been a certain amount of confusion because of the many attempts to correlate rigidly adult structures with those found at an early stage in the development of the embryo. Thus epithelium has been equated with ectoderm and endoderm and connective tissue with mesoderm. At the same time it has become quite clear that some structures such as kidney and adrenal gland and probably liver are mesodermal in origin, and yet structurally their parenchymal components conform with the general idea of " epithelial " cells. It is for this reason that the term " parenchyma " was used earlier.

TABLE I.—*Results Obtained After Staining Different Normal and Non-malignant Tissues with Fluorescein-protein Complexes*

Comparable results were found in all cases namely, the cytoplasm of all epithelial cells fluoresced well. The number of observations made is greatly in excess of this total since many tissues were examined by a battery of protein conjugates.

| Tissue | Condition | Number of cases |
|---|---|---|
| Epidermis | (*a*) Normal epidermis (man, mouse, rat, rabbit) | 62 |
| | (*b*) Hyperkeratotic wart (man) | 8 |
| | (*c*) Molluscum pseudocarcinomatosum (man) | 12 |
| | (*d*) Irritated epithelium (man) | 12 |
| | (*e*) Sudoriferous adenoma (man) | 1 |
| Breast | (*a*) Lactating breast (mouse) | 4 |
| | (*b*) Hyperplasia (man) | 62 |
| | (*c*) Fibroadenoma (man) | 14 |
| Stomach | (*a*) Normal mucosa (man) | 22 |
| Colon | (*a*) Normal mucosa (man, mouse, fowl, rat, lizard, frog, sheep axolotl) | 50 |
| | (*b*) Polyps (man) | 6 |
| Lung | (*a*) Bronchial mucosa (man, mouse) | 20 |
| | (*b*) Bronchial adenoma (man) | 1 |
| Thyroid gland | (*a*) Normal thyroid (man) | 38 |
| | (*b*) Thyrotoxicosis (man) | 22 |
| | (*c*) Colloid goitre (man) | 26 |
| | (*d*) Foetal adenoma (man) | 15 |
| Lymph node | (*a*) Normal (man, mouse, rabbit) | 28 |
| Liver | (*a*) Normal (man, mouse, rat, rabbit, axolotl, frog, fowl, sheep) | 55 |
| Kidney | (*a*) Normal (man, mouse, rabbit, rat, fowl) | 16 |
| Blood | (*a*) W.B.C. (man) | 197 |
| | (*b*) W.B.C. (mouse) | 160 |
| | (*c*) W.B.C. (fowl, ox, sheep, frog, axolotl) | 15 |
| | (*d*) R.B.C. nuclei (fowl, frog, axolotl, fish, lizard) | 12 |
| | (*e*) Chronic leukaemia (man) | 166 |
| | (*f*) Chronic leukaemia (mouse) | 4 |
| Endometrium and cervix | (*a*) Hyperplasia (man) | 6 |
| | (*b*) Endometriosis (man) | 4 |
| | (*c*) Cervical erosion (man) | 7 |
| | Total | 1045 |

Nevertheless the distinction in adult tissues is commonly accepted as being between epithelium and connective tissue. Usually epithelium is composed of cells with a considerable amount of cytoplasm surrounding a well-defined nucleus and the individual cells are arranged in groups with a very small amount of intercellular cement or material. Viewed from this point of view it is of little moment what was the remote origin of the cells. Connective tissues on the other hand are characterized, from the very nature of their function, by a considerable amount of intercellular material and the cell itself is often either small or may be difficult to recognize, the position of the cell being indicated by the nucleus.

The observations therefore, in the employment of the fluorescein-globulin stain, are that epithelial cells in normal circumstances stain well, but connective tissue cells usually fail to do so. Occasionally groups of connective tissue cells do stain and, in such cases, these stand out clearly from the remainder of the connective tissue just as epithelial cells do.

TABLE II.—*Results Obtained by Staining Various Neoplastic Tissues with Different Fluorescein-protein Complexes*

In all cases examined identical differential staining was obtained—normal tissue fluoresced but the malignant counterparts failed to do so.

| Tissue | Condition | Number of cases |
|---|---|---|
| Epidermis | (*a*) Squamous cell carcinoma (man) | 15 |
| | (*b*) Basal cell carcinoma (man) | 4 |
| | (*c*) Induced carcinoma (mouse) | 6 |
| Breast | (*a*) Carcinoma (man) | 34 |
| | (*b*) Spontaneous carcinoma (mouse) | 18 |
| | (*c*) Transplanted carcinoma (mouse) | 14 |
| | (*d*) Walker 256 carcinoma (rat) | 7 |
| Stomach | (*a*) Carcinoma (liver) | 13 |
| Colon | (*a*) Carcinoma (man) | 28 |
| Lung | (*a*) Carcinoma (man) | 9 |
| Thyroid | (*a*) Carcinoma (man) | 3 |
| Lymph node | (*a*) Secondary Melanoma (man) | 3 |
| | (*b*) Secondary carcinoma (man) | 7 |
| Liver | (*a*) Hepatoma (man) | 3 |
| | (*b*) Cholangioma (man) | 1 |
| | (*c*) Hepatoma (rat) | 18 |
| | (*d*) Cholangioma (rat) | 8 |
| | (*e*) Secondary carcinoma (ox) | 1 |
| | (*f*) Secondary carcinoma (pig) | 1 |
| Kidney | (*a*) Carcinoma (man) | 3 |
| Adrenal | (*a*) Carcinoma (ox) | 1 |
| Blood | (*a*) Acute leukaemia (man) | 19 |
| | (*b*) Acute leukaemia (mouse) | 60 |
| | (*c*) Leucosis (fowl) | 1 |
| | (*d*) Leukaemia (sheep) | 3 |
| | (*e*) Leukaemia (ox) | 1 |
| | Total | 281 |

In this differentiation of epithelium from ordinary connective tissue the same kind of technical approach as is employed with all types of stain must be adopted. There is an optimum time for staining in order to provide the most satisfactory delineation of the various structures being examined. Obviously if the stain is applied for too short a time tissues will not stain. On the other hand if the stain is allowed to act for too long a period, overstaining of the tissue occurs and even those which do not ordinarily take up some of the stain may do so ; thus a degree of differentiation which will allow the distinction of the various parts cannot be obtained. To this extent times of staining and concentration of stains must be arbitrary, but it is no more arbitrary than is the case with every other stain that is employed in histological work.

The essential component of the stain used is the protein ; since this is not ordinarily demonstrable visually it is conjugated with a dye. The concentration of material obtained in a cell is so small that most dyes employable in this way are not sufficiently easily recognizable to make the observations easy, rapid and certain. It is for this reason that the fluorescing dyes have been used—and this adds some further complications.

The observations are made on the effect of incident ultra-violet light so that a fluorescence is observed. The colour of this fluorescence depends on the dye used and in the one most commonly used—fluorescein—the fluorescence is of a green colour ; when rhodamine is used the result is orange—and still others may be employed. Some of the tissues in the body have a natural fluorescence (in ultra-violet light) which is independent of the staining, and although this may be prominent, because of the difference in colour of the fluorescence it should not present any significant problem ; it is important that this auto-fluorescence should be thoroughly appreciated. Not only does the difference in colour of the fluorescence light indicate the nature of this phenomenon, but the distribution of the fluorescing material clearly indicates that, in its morphological arrangement, it is different and distinct from the cells of the tissue.

The important connective tissue component which fluoresces naturally is elastic tissue. This has a linear distribution and occurs characteristically in some situation such as the walls of small blood vessels (Fig. 7). In some such cases its site and general form make its diagnosis easy thus indicating the nature of other masses of elastic tissue which may not be so characteristically disposed and, furthermore, it often helps in recognition of various parts of the tissues apart from staining. The elastic tissue fluoresces a yellow colour which is quite distinct from the green of the fluorescein-globulin stain. Some of the mucins, although they fluoresce well because they stain with the fluorescein-globulin, sometimes show a small degree of a bluish fluorescence. This applies to the mucins of both epithelial and connective tissue origin. Although not of connective tissue, it might be mentioned here that keratin also has a bright blue-white auto-fluorescence (Fig. 6). In all such cases the fluorescence is completely independent of the phenomena being discussed here, but must be clearly recognized in all applicable tissues so that it may be excluded from consideration of the fluorescence due to the presence of the stain.

The connective tissue cells which stain with the fluorescein-globulin have been principally histiocytes, plasma cells, enlarged endothelial cells and foreign body giant cells (Fig. 13, 14). In general, the small round cells of the lymphocyte type do not obviously fluoresce, but the larger cells—the macrophages—fluoresce brightly ; this is particularly apparent when these occur in groups. In addition to these mononuclear cells, polymorphonuclear leucocytes also stain (Fig. 1, 2).

Examination of blood films shows that in normal circumstances red blood corpuscles do not stain but cells of the white series of all kinds do (Louis, 1957*d*) and this applies also to the mononuclear cells of the blood including the small round cells (Fig. 1, 2). The lymphocytes require further study because of some apparent discrepancy between those in the blood stream and in the tissues. The apparent difference may not necessarily be a real one but may well be due to the relative difficulty in demonstrating fluorescence of the small amount of material in sections as compared with the relative ease of observing such changes in a film.

Occasionally some cells which might be expected to stain (or which, in other circumstances, are found to stain well) fail to do so. It is well known that fluorescence of tissues may be damped down or obscured by specific materials present. This may be due to some physico-chemical change induced in the material which previously fluoresced, or it may be a simple physical blanketing of the fluorescent material. A good example of this is some of the cells containing melanin pigment (Fig. 6). Most of the pigment-producing and pigment-containing cells in the skin fluoresce well, but occasionally some of these do not and it is found that this is associated with the presence of a relatively larger amount of pigment in the cells.

In these cases this lack of staining may be due to displacement of protoplasm by a product of tissue activity, or to an obscuring of the fluorescence phenomenon which is normally present. Investigations of cells from which the pigment has been removed chemically have not yet been made.

The problem, therefore, is one of the mechanism of staining. It is clearly not solely a matter of the type or origin of the cell. The one feature which appears to be common to the cells which stain is that they possess a considerable mass of cytoplasm. It is of no consequence whether the cells are epithelial or of connective tissue origin ; provided that there is a sufficient volume of cellular material this, in ordinary circumstances, will stain. If the amount of cytoplasm is small then the staining of any such material may not be recognized.

A point of interest here is the staining of endometrial tissue. The glands stain well in the normal fashion, that is, the cytoplasm stains though the nuclei do not but, in addition, the cells of the stroma stain similarly (Fig. 9, 10). This phenomenon may be considered from two points of view. In the first place the stroma cells are quantitatively similar to the cells of the glands, that is, they contain a considerable amount of cytoplasm, but there is also the question of the relation between the two kinds of cells. It has been frequently stated that the glands are " epithelial " whereas the stroma is connective tissue. From the purely morphological point of view this is true, but the implication that they are therefore different from each other is quite unjustified, and recent work has indicated that stromal cells can develop from glands, and glands grow and differentiate from stroma (Brines and Blain, 1943). It is unnecessary to discuss this point in detail here but it is clear that cells of different forms may show similar staining characteristics provided that they are similarly endowed with cytoplasm.

In all these cases it is the protoplasm of the cells which stain and the nuclei do not. This is very clearly seen in photomicrographs of, for example, the liver where the cells show a brightly fluorescing cytoplasm and an ovoid or round area of absence of fluorescence corresponding to the position of the nucleus (Fig. 5). In the case of red blood cells, however, the situation is reversed (Louis, 1958*a*). The haemoglobinized red cell does not stain but, in the lower vertebrates where all the red corpuscles are nucleated, the nuclei fluoresce brightly (Fig. 3). This kind of phenomenon would present considerable interpretative difficulties with the older idea of a serological reaction and antigen-antibody phenomenon but, in the light of the present concept, it would appear that in the nuclei of the red cells there must be some basic protein which will unite with the relatively acid fluorescein-globulin complex.

The failure of the red corpuscles to stain in normal circumstances presents, at first sight, a problem in that there is in the cells a considerable mass of material ; but at the same time there is relatively little cytoplasm comparable with that seen in other cells since haemoglobin is the predominant constituent and the isoelectric point of oxyhaemoglobin is below the pH at which staining is carried out. Since at this pH fluorescein-globulin complex and haemoglobin would have like charges, mutual repulsion rather than attraction of the two charged components could be expected.

The complexity of the problems is shown by the fluorescence of red blood corpuscles in certain circumstances. This occurs particularly in cases in which there appears to be some serological change of the kind seen in antigen-antibody phenomena (Louis, 1957*d* ; Hughes 1958*b*). Thus it is found in cases of acquired haemolytic anaemia and in lupus erythematosus (Fig. 4). Observations have been made which indicate that, in this case, the phenomenon is one on the surface of the cell due to coating of the cell with protein which, in its turn, unites with the stain. This is quite a different phenomenon from the staining of the cytoplasm of cells of other tissues.

That the cells and tissues of the central nervous system failed to stain requires elucidation. This peculiarity was not unexpected in view of other features of staining of nervous tissue. The large amount of lipoid material in this tissue is doubtless significant as is readily recognized by all who have worked, in even a small way, with lipoproteins. Furthermore there is the observation that, in experiments carried out with insulin tagged with fluorescein, this has been shown to involve all cells with the exception of red blood corpuscles and cells of the central nervous system.

These cells therefore can be regarded as a special group which differ physically and chemically from those of other tissues in the body.

---

### EXPLANATION OF PLATES

FIG. 1.—Fluorescence photomicrograph of a peripheral blood film (man) stained with fluorescein-protein complex and showing three fluorescing white cells. The red blood cells do not fluoresce. × 120.

FIG. 2.—Same area as shown in Fig. 1 subsequently stained by Leishmann's method for comparison.

FIG. 3.—Fluorescence photomicrograph of a peripheral blood film (fowl) stained with fluorescein-protein complex and showing one fluorescing white cell (below) and nuclei of red cells. × 160.

FIG. 4.—Fluorescence photomicrograph of a peripheral blood film taken from a patient with acquired haemolytic anaemia (positive Coombs test). All the red blood cells fluoresce. × 200.

FIG. 5.—Fluorescence photomicrograph of a section of rat liver stained with fluorescein-protein complex and showing a bright and uniform fluorescence of the cytoplasm of all the liver cells. The nuclei do not fluoresce. × 160.

FIG. 6.—Fluorescence photomicrograph of a section of a simple mole (man) stained with fluorescein-protein complex. The cells containing pigment absorb the fluorescence and thus do not become visible. The linear fluorescence beneath the epidermis is an auto-fluorescence (which was yellow as opposed to the green colour of the main tissue) due to elastic tissue. × 160.

FIG. 7.—Fluorescence photomicrograph of a section of mouse kidney stained with fluorescein-protein complex and showing fluorescence of the cytoplasm of the tubular cells. Note the linear (yellow) auto-fluorescence of the internal elastic lamina of the arteriole. × 240.

FIG. 8.—Same area as shown in Fig. 7 subsequently fixed in formol-saline and stained with haematoxylin and eosin for comparison. × 240.

FIG. 9.—Fluorescence photomicrograph of a section of endometrial scrapings stained with fluorescein-protein complex and showing fluorescence of both stromal and glandular cells. The vacuoles of the cells and the nuclear material take the place of cytoplasm and thus results in diminution of the brightness of staining. × 160.

FIG. 10.—Same area as shown in Fig. 9 subsequently fixed in formol-saline and stained with haematoxylin and eosin for comparison. × 160.

FIG. 11.—Fluorescence photomicrograph of a section of a transplanted carcinoma of the breast (mouse) which is sharply demarcated from an area containing many plasma cells, macrophages and polymorphonuclear leucocytes. The inflammatory cells fluoresce brightly but the tumour tissue below fails to do so. × 320.

FIG. 12.—Same area as shown in Fig. 11 subsequently fixed in formol-saline and stained with haematoxylin and eosin to show the structure of the non-fluorescing area. A capillary vessel cut longitudinally separates the non-malignant from the malignant cells. × 320.

FIG. 13.—Fluorescence photomicrograph of a section of a spontaneous carcinoma of the breast from a lactating mouse. The overlying skin, glandular tissue and some striated muscle fibres fluoresce brightly but the tumour tissue below fails to do so. × 160.

FIG. 14.—Same area as shown in Fig. 13 subsequently fixed in formol-saline and stained with haematoxylin and eosin to show the structure of the non-fluorescing area. × 160.

1

2

3

4

5

6

Louis.

7

8

9

10

11

12

13

14

In resumé, therefore, a considerable proportion of the cells of the body stain with fluorescein-globulin in normal circumstances. These include all the cells of epithelium or parenchymal tissues, all connective tissue cells which have well-defined cytoplasm and this includes particularly the histiocytes and wandering cells, and the white blood corpuscles of the bone marrow and blood. The staining of all these cells occurs in the cytoplasm and their nuclei do not stain ; this staining depends on the presence in the cytoplasm of a basic protein which unites with the relatively acidic fluorescein-protein. The nuclei of nucleated red cells (particularly in the lower vertebrates) also stain and this phenomenon has not yet been elucidated.

There are certain circumstances in which normal cells do not stain. Ordinary connective tissue in which there is a very small amount of cytoplasm in relation to the connective tissue nuclei fails to show any significant staining and, indeed, from the point of view of ordinary routine examination such connective tissue can be said not to stain. There are circumstances in which the cytoplasm is replaced or obscured by intracellular components, a particularly good example being the occurrence of melanin in pigment-producing cells and these cells will not stain.

It can be said, therefore, that all the normal cells of the body which contain a significant amount of cytoplasm, with the exception of certain well-defined examples (the reason for their differences being susceptible to simple explanation) stain with these fluorescein-protein stains in a similar manner.

When malignancy occurs there are demonstrable changes in the cells, one of which is the loss of a basic protein in the cytoplasm and thus these tissues fail to stain (Fig. 11, 12, 13, 14). This constitutes a striking phenomenon, particularly when portion of a tumour and some of the normal tissue of the area are present in the same section.

It is obvious that, in view of discrepancies in the assumption that staining of normal tissues is universal, all cases of non-staining tissue, assumed to be malignant, should be compared with a normal tissue of a comparable type. Such comparison is readily made in epithelial tumours and in those of tumours containing cells with a considerable amount of protoplasm.

In the case of the sarcomata, however, observations are not so significant. There is no question but that the sarcomata do not stain, but then original cells often fail to stain, but this is because they do not possess enough cytoplasm.

If we consider some of the reticulosarcomata where the prototype is a cell which has a moderate amount of protoplasm and which, therefore, ordinarily stains, we have a relationship similar to that seen in the case of the epithelial growths. Thus, though the method cannot be used with any degree of certainty, in the case of the sarcomata in general, if the tumour cells are relatively large and do contain cytoplasm, the demonstration of failure to stain has considerable significance ; however, here the conclusions are drawn from a general consideration of the characteristics of cytoplasm of cells rather than by a direct comparison between malignant tumours and the corresponding normal tissue.

Thus, when changes occur in connective tissue so that the cells become larger, for example, when fixed cells change their character and become well-defined bodies (wandering cells of the histiocyte type), they develop a clearly defined staining capacity, whereas when the change is in the direction of malignancy such staining capacity does not appear. Doubtless in due course, with improved and more refined methods of staining, it may be possible to demonstrate some staining character in the connective tissue cells of the normal tissues.

A problem which is related to the above question, but which is on a less general plane, is that of the significance and relation to malignant tissues of hyperplastic tissues and innocent tumours. This will be the subject of a subsequent paper.

### SUMMARY

Fluorescein-serum protein may be used as a serologically non-specific stain which stains normal but not malignant tissues.

All normal tissues of vertebrates (and some invertebrates) stain well and fluoresce brightly provided there is a sufficient amount of protoplasm in the cells.

This phenomenon is due to the presence in the normal cell of a protein complex whose isoelectric point is sufficiently different from that of the serum proteins to allow protein-protein interaction.

Normal cells which fail to stain are :

(i) Resting connective tissue cells which have considerable intercellular substance but little cellular proteoplasm, such as a normal fibroblast ;

(ii) cells containing materials such as melanin pigment which obscures the protoplasm of the cell ;

(iii) red corpuscles where haemoglobin (the isoelectric point of which is too near that of the proteins used in the stain to allow combination) constitutes a major part of the cell material.

In all cases malignant tissue fails to stain. The significant feature is that the cells of such a tumour contain cells with, often, voluminous protoplasm but this does not show the phenomenon.

The conclusion that non-malignant cells may be distinguished from normal cells has been demonstrated without real exceptions (excluding the normal haemoglobinized red cell). Scrutiny of the few exceptions indicates that the differences are of a form and degree (as indicated by the lymphocyte) that will be resolved with improvements in technique.

(iv) Cells of the central nervous system which can be regarded as constituting a special group.

This work was supported by a grant from the Anti-Cancer Council of Victoria.

### REFERENCES

Brines, O. A. and Blain, J. H.—(1943) *Surg. Gynec. Obstet.*, **76**, 197.

Coons, A. H. and Kaplan, M. H.—(1950) *J. exp. Med.*, **91**, 1.

Hughes, P. E.—(1957) *Aust. N.Z. J. Surg.*, **26**, 302.—(1958*a*) *Cancer Res.*, **18**, 426.—(1958*b*) *Aust. Ann. Med.*, **7**, 228.

*Idem*, Louis, C. J., Dineen, J. K. and Spector, W. G.—(1957) *Nature*, **180**, 289.

King, E. S. J., Hughes, P. E. and Louis, C. J.—(1958*a*) *Brit. J. Cancer*, **12**, 5.—(1958*b*) *Cancer*, in press.

Louis, C. J.—(1957*a*) *Stain Tech.*, **32**, 279.—(1957*b*) *Aust. Ann. Med.*, **6**, 277.—(1957*c*) *Aust. N.Z. J. Surg.*, **27**, 146.—(1957*d*) *Aust. Ann. Med.*, **6**, 300.—(1958*a*) *Ibid.*, **7**, 219.—(1958*b*) *Brit. J. Surg.*, in press.—(1958*c*) *Surg. Gynec. Obstet.*, **107**, 317.

Sorof, S. and Cohen, P. P.—(1951) *Cancer Res.*, **11**, 283.

Weiler, E.—(1952) *Z. Naturf.*, **7**, 324.—(1956*a*) *Ibid.*, **11**b, 31.—(1956*b*) *Brit. J. Cancer*, **10**, 553.